# Supplementary material for: Persistent Lipid Accumulation Leads to Persistent Exacerbation of Endoplasmic Reticulum Stress and Inflammation in Progressive NASH via the IRE1α/TRAF2 Complex
Source: Molecules. 2023 Apr 3;28(7):3185. doi: 10.3390/molecules28073185 (PMC10095702; doi:10.3390/molecules28073185)
Supplement: Supplementary file 1 [file molecules-28-03185-s001.zip › molecules-2219299-supplementary.pdf]

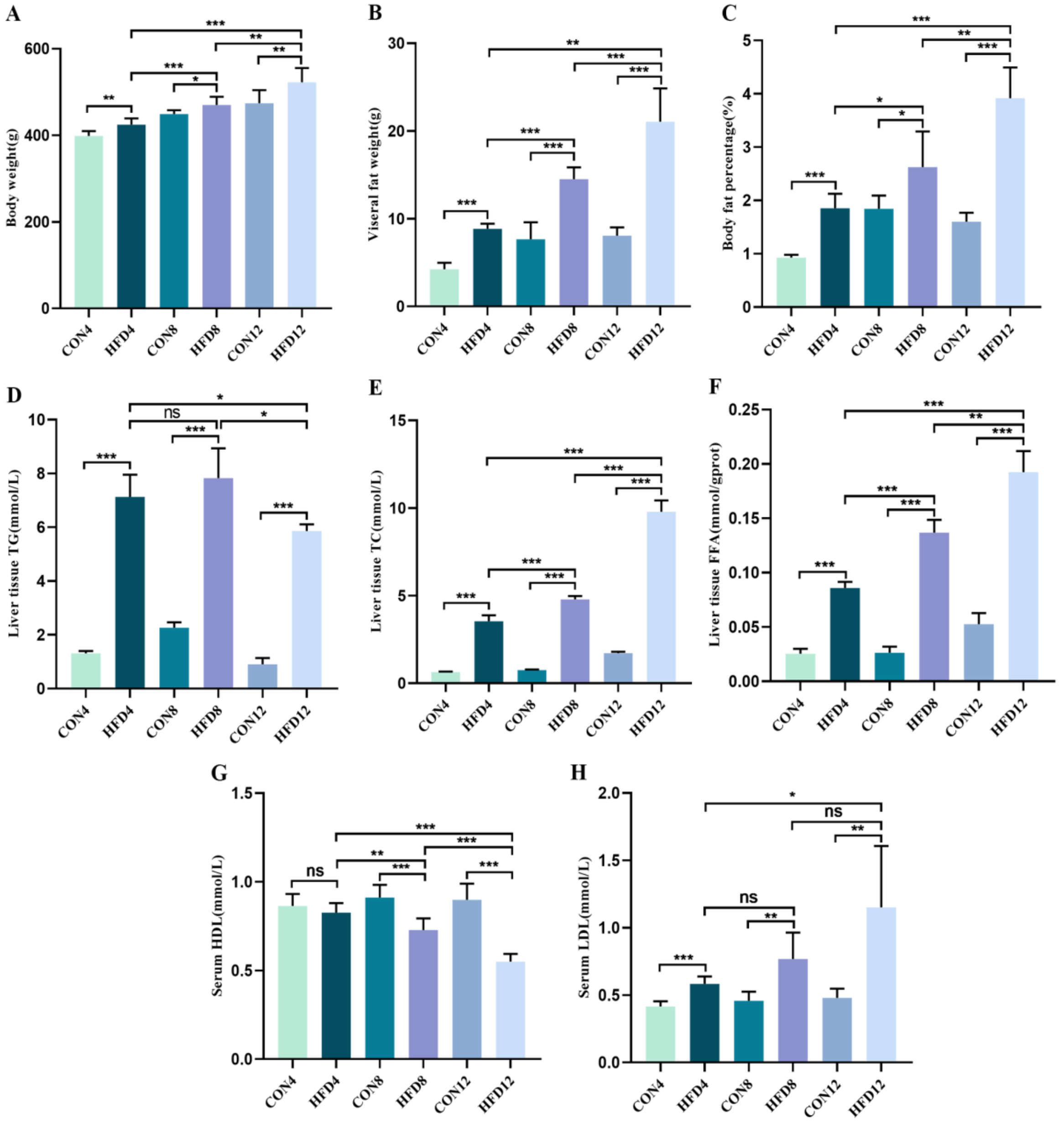

Figure S1. Detection of body weight, visceral fat weight, body fat percentage, and blood lipids-related indicators. (A-C) Body weight, visceral fat weight, and body fat percentage ($n \geq 6$). (D-F) Triglyceride (TG), total cholesterol (TC), and free fatty acid (FFA) in liver tissue ($n \geq 6$). (G-H) High-density lipoprotein (HDL) and low-density lipoprotein (LDL) in serum ($n \geq 6$). Student's *t*-test was used to compare differences between two groups. One-way analysis of variance (ANOVA) was applied to compare differences between three groups. * $p < 0.05$, ** $p < 0.01$ and *** $p < 0.001$. ns, not significant.

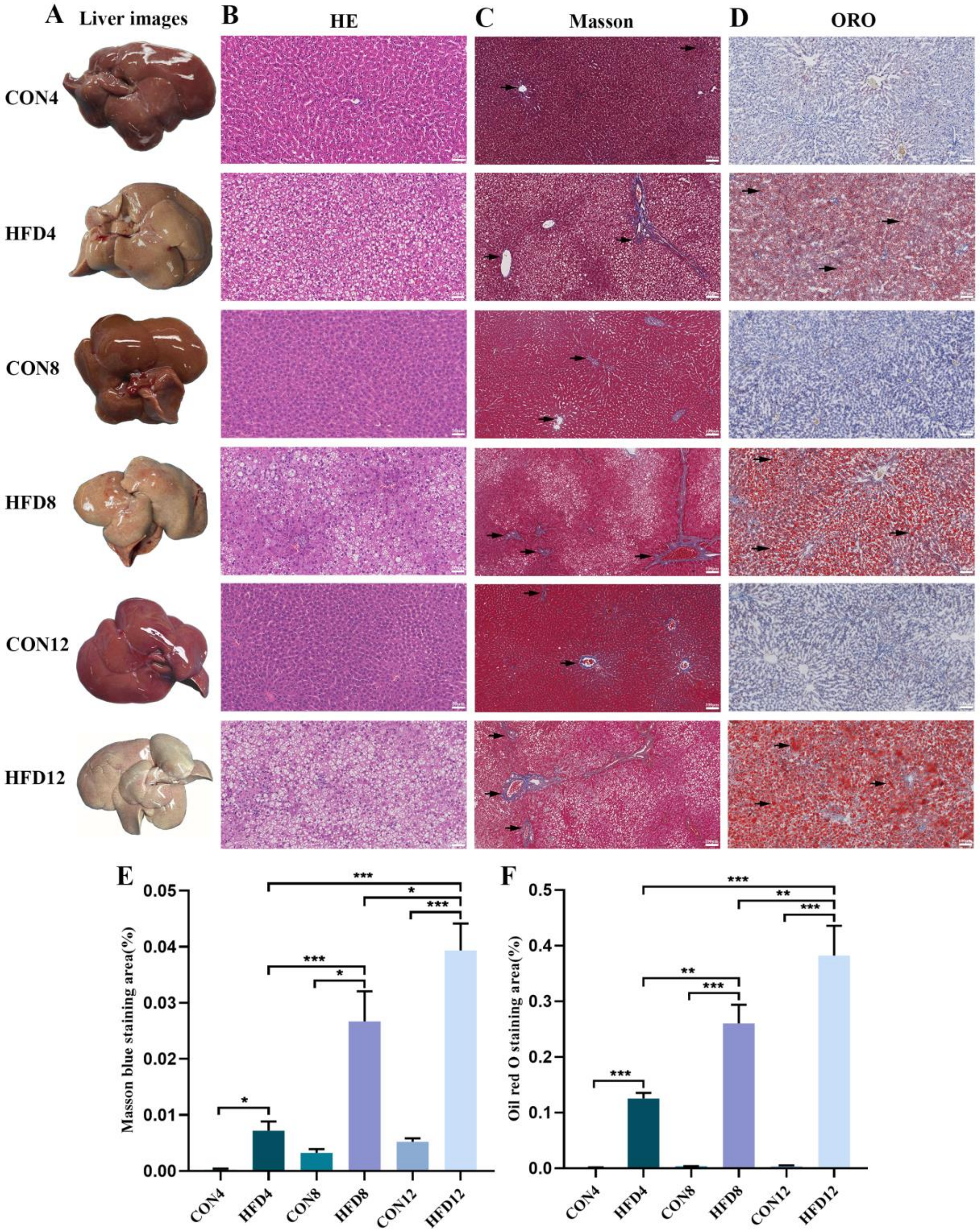

Figure S2. Detection of histological staining. (A) Liver images. (B) HE staining (scale bar = 200 μm). (C) Masson staining (scale bar = 100 μm), the blue staining indicated by the arrow represents collagen fibers. (D) ORO staining (scale bar = 100 μm), the red staining indicated by the arrow represents lipid droplets. (E,F) Quantification of collagen fibers and lipid droplets ($n = 3$). Student's t-test was used to compare differences between two groups. One-way analysis of variance (ANOVA) was applied to compare differences between three groups. * $p < 0.05$, ** $p < 0.01$ and *** $p < 0.001$.

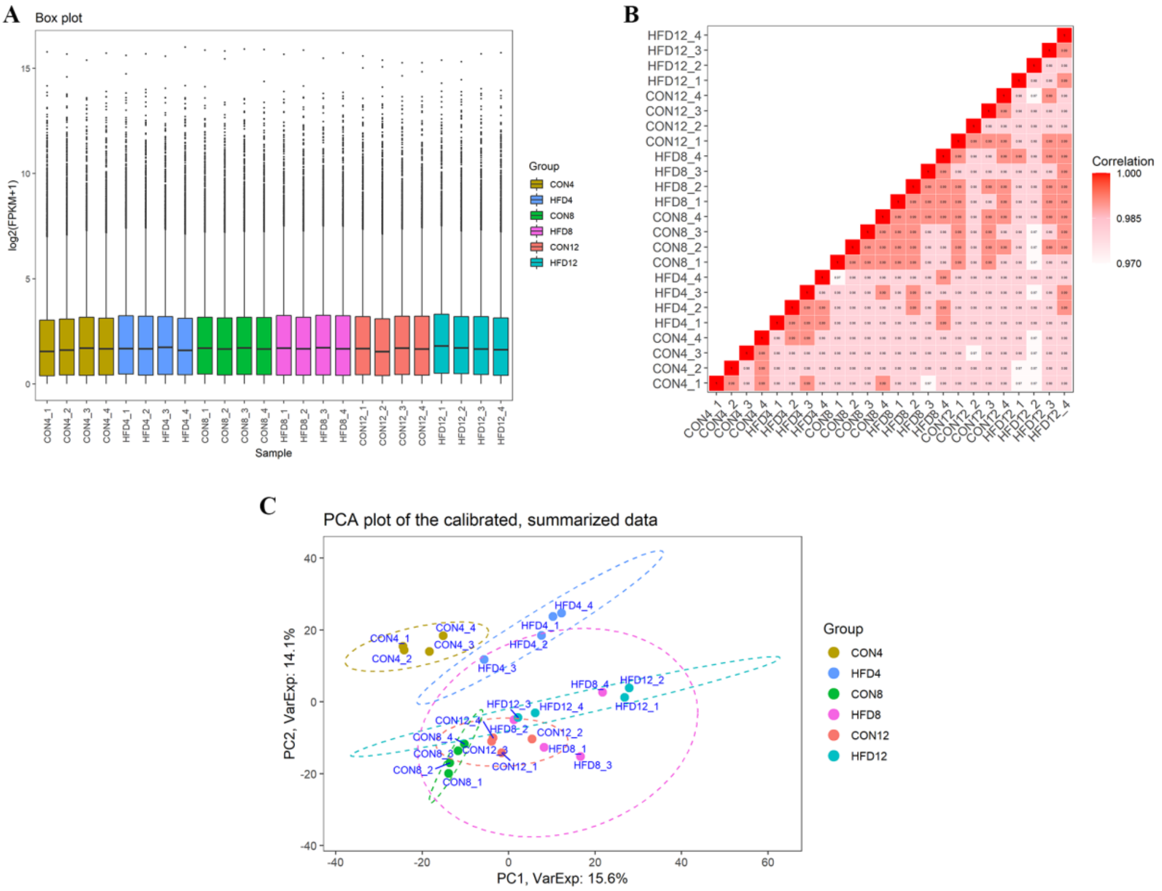

Figure S3. The sample genes expression, samples correlation analysis, and principal components analysis.

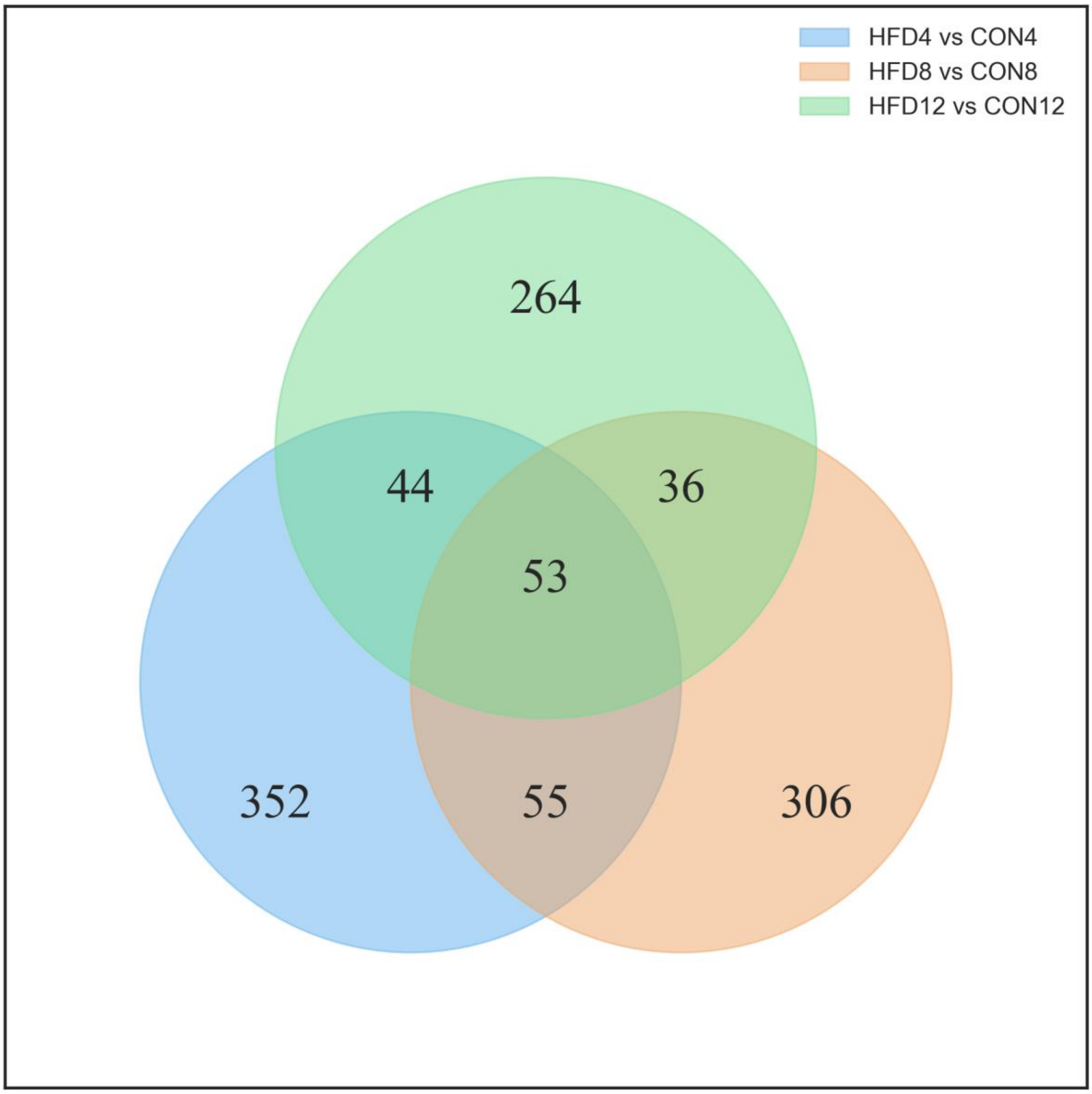

Figure S4. The number of overlap genes in different comparisons (Venn).
